# Yellow Mealworm (*Tenebrio molitor*) Powder Promotes a High Bioaccessible Protein Fraction and Low Glycaemic Index in Biscuits

**DOI:** 10.3390/nu15040997

**Published:** 2023-02-16

**Authors:** Anca Mihaly Cozmuta, Alexandra Uivarasan, Anca Peter, Camelia Nicula, Dalma Emoke Kovacs, Leonard Mihaly Cozmuta

**Affiliations:** 1 Technical University of Cluj Napoca, North University Center of Baia Mare, Victoriei Str. 76, 430122 Baia Mare, Romania; 2 Research Institute for Analytical Instrumentation, INCDO INOE 2000, Donath 63, 400293 Cluj Napoca, Romania

**Keywords:** functional biscuits, slowly digestible starch, rapidly digestible starch, in vitro digestion modelling

## Abstract

Traditional biscuits are considered products with poor nutritional value because of their large share of rapidly digested starch, which results in an elevated glycaemic index. This paper explores the improvement of the nutritional value of biscuits by adding yellow mealworm (Tenebrio molitor) powder. Four biscuit recipes containing 0%(R1), 10%(R2), 15%(R3), and 20%(R4) of yellow mealworm powder were prepared and subjected to sensorial analysis. The R3 biscuits were selected for further investigation, as they had the highest acceptability. Compared to the reference R1, the R3 biscuits showed an improved nutritional profile in terms of protein, fat, ash, minerals, fibres, essential amino acids, and unsaturated fatty acids, and lower amounts of carbohydrates and 5-hydroxymethylfurfural. The in vitro protein digestibility in R3 improved 1.12-fold compared to R1. No significant difference was found between the digestibility of the lipids released from R1 and R3. A higher fraction of slowly digestible starch was present in R3 compared to R1. The starch digestibility and estimated glycaemic index were 72.96% and 79.56% in R3, which can be compared to 78.79% and 90.14%, respectively, in R1. Due to their enhanced nutritional profile, higher bioaccessible protein fraction, and lower glycaemic index, yellow mealworm powder biscuits can be considered a more nutritious alternative to traditional biscuits.

## 1. Introduction

Biscuits are a ready-to-eat bakery product with a long shelf-life, and are traditionally prepared with wheat flour, honey, sugar, and fat. The high sugar content of biscuits makes them highly energetic products that contain a large amount of rapidly digested starch, which results in an elevated glycaemic index during digestion. Similar to honey or sugar, the high fat content of biscuits also gives them a high energy value [1]. The poor nutritional value of traditional biscuits is associated with the risk of developing metabolic or cardiovascular diseases [2]. Improving the nutritional value of biscuits for a healthy lifestyle is often achieved by the addition of fibre [3], proteins [4], or legume flour [1,5]. A recently explored solution to enhancing the nutritional value of biscuits is the addition of edible insect powders. The positive assessment recently issued by the European Food Safety Authority (EFSA) concerning the consumption of house crickets, yellow mealworms, and grasshoppers as novel foods [6] has offered new opportunities for developing functional food. The increasing demand for high-protein, low-fat, and economical food sources, along with shifting trends in dietary needs, is likely to stimulate the edible insect market. The edible insect market is growing; it exceeded USD 112 million globally in 2019 and is forecast to grow at a compound annual growth rate of over 47% between 2019 and 2026 [7]. Due to their high bioactive compound content, which includes essential amino acids, unsaturated fatty acids, and fibre, as well as their low carbohydrate content [8,9], insect powders may be a solution to alleviating the nutritional imbalance of traditional biscuits.

The beneficial health-related results of adding insect powder to bakery products have been presented in the literature. As shown in the study conducted by Mihaly Cozmuta et al. [10], the addition of 10% cricket and yellow mealworm powders (YMP) in bread promoted higher bioaccessible fractions of Na, K, Ca, Mg, P, Fe, Zn, Mn, and Li compared to conventional bread containing 100% white flour. Di Mattia et al. [11] have demonstrated that the water-soluble extracts of silkworms, crickets, and grasshoppers display five-fold higher antioxidant capacity values than fresh orange juice. Zielinska et al. [9] have reported increased total phenolic content and antioxidant capacity in muffins fortified with 10% yellow mealworm (*Tenebrio molitor*) powder, as well as a lowered carbohydrate content and glycaemic index, compared to the control muffins made entirely of wheat flour. The rapidly digested starch content of shortcake biscuits decreased in parallel with the increase in slowly digested starch after the addition of *Tenebrio molitor* flour [12]. Bas et al. [13] have estimated that the glycaemic indices of standard and cricket biscuits are 49.28 and 47.84, respectively, while the in vitro protein digestibility of cricket biscuits (45.19%) was almost two times that of standard biscuits (26.47%). The new biscuits had similar sensory properties to traditional ones and the advantage of improved nutritional value.

Since the availability of biscuits with improved nutritional value as well as similar taste and texture to traditional biscuits is of great interest, this study aimed to prepare biscuits fortified with yellow mealworm (*Tenebrio molitor*) powder (YMP) and assess their nutritional value. The study is organised as follows: (i) a comparative evaluation of the nutritional value of white wheat flour and yellow mealworm powder is presented; (ii) the preparation of biscuits carried out by replacing white wheat flour with 10%, 15%, and 20% yellow mealworm powder, and reference biscuits made entirely of white wheat flour, is described, and all of these samples were subjected to sensorial study; and (iii) the selection of the biscuits that received the highest score for the overall acceptability attribute is reported, along with an investigation of their nutritional value compared to the reference biscuits.

## 2. Materials and Methods

### 2.1. Raw Materials and Reagents

The ingredients used in biscuits’ preparation were white wheat flour (Baneasa, Romania), yellow mealworm (*Tenebrio molitor*) powder (Exotic K, Romania), powdered bicarbonate of soda (Dr. Oetker, Germany), honey (Roua Florilor, Romania), soy milk (Alpro, Belgium), sunflower oil (Unisol, Bunge Romania), salt (Olympia, Romania), and vanilla sugar (Dr. Oetker, Germany). The following reagents, which were used for chemical investigations, were purchased from Merck (USA): KOH, HCl, petroleum ether, HNO_3_, isooctane, NaHSO_4_, FAME 37, NH_4_OH, acetyl chloride n-butanol, trifluoroacetic anhydride, Carrez I, Carrez II, *p*-toluidine, barbituric acid, KCl, KH_2_PO_4_, NaHCO_3_, NaCl, MgCl_2_·6H_2_O, (NH_4_)_2_CO_3_, CaCl_2_·2H_2_O, salivary amylase, pepsin, gastric lipase, bile salts, trypsin, D-glucose, HMF (purity ≥ 99%), FAME Mix 37 (CRM—certified reference materials), and Amino acid (acidic and neutral) standard (±4%). Ultrapure water (0.05 μS cm^−1^) produced from Millipore (Millipore, Bedford, MA, USA) was used to prepare solutions.

### 2.2. Characterisation of White Wheat Flour (WWF) and Yellow Mealworm (Tenebrio molitor) Powder (YMP)

#### 2.2.1. Moisture Content

The moisture content was determined gravimetrically [14] by drying (Binder GmbH, Germany) the homogenised samples at 105 °C for 4 h until obtaining a constant weight.

#### 2.2.2. Ash Content and Speciation of Mineral Elements

The total mineral content was obtained by incinerating the samples for 24 h at 550 °C in a muffle furnace (Nabertherm, Germany), and is expressed in g of ash/100 g of dry sample [15].

The speciation of mineral elements involved microwave digestion [16], and the content was quantified using a Perkin Elmer AAnalyst 800 spectrometer (Perkin Elmer, USA). The results are expressed in mg of mineral/100 g dry sample.

#### 2.2.3. Total Protein and Amino Acid Profile

The Kjeldahl assay (Velp Scientifica UDK 127, Italy) [17] was employed to measure the total protein content. The results were calculated according to Equation (1):

Protein (g/100 g on dry weight basis) = TN × 6.25,(1)

where TN is the total nitrogen content (%), and 6.25 is the conversion factor.

The water-soluble amino acid profile was investigated by hydrolysing and derivatising the samples before injection onto a gas chromatograph with flame ionization detector GC-FID Trace GC1310 (Thermo Scientific, USA). A total of 1 g of the sample was ground, rigorously mixed with 5 mL of distilled water [18], and centrifuged for 5 min. A volume of 0.5 mL of supernatant was harvested and passed through a Dowex 50W-W8 ion exchange resin and, subsequently, eluted with a 4M NH_4_OH solution. Derivatisation was performed in two steps: esterification of the extracted amino acids with a mixture consisting of acetyl chloride n-butanol (4:1 *v*/*v*) for 1 h at 100 °C, and acetylation with 100 μL of trifloractic anhydride at 60 °C for 30 min. A volume of 1 μL of the solution of the derived amino acids was separated into a chromatograph gas with a flame ionization detector GC-FID Trace GC1310 (Thermo Scientific) using a capillary column Rtx-5MS capillary 30 mm × 0.25 mm at a film thickness of 0.25 μm with a schedule of temperature increase from 50 °C (1 min) by 10 °C · min^−1^ to 100 °C, 4 °C · min^−1^ at 200 °C, and 20 °C · min^−1^ at 290 °C (maintained for 5 min). The injector was kept at 250 °C and the detector at 280 °C. The carrier gas was He, with a flow rate of 1 mL · min^−1^. The external calibration was carried out using standard solutions obtained from seven serial dilutions of a working solution containing mixtures of pure analytical standards. Accuracy and precision were assessed by replicate analysis of standards at three concentration levels and replicate analysis of spiked samples at three concentration levels (low, medium, and high) on three validation days. Precision was estimated as the percentage of relative standard deviation (RSD) of replicate standards within one validation batch (intra-day) and between validation batches (inter-day). The results are expressed as the amino acid percentage of the total amino acids.

#### 2.2.4. Total Lipid and Fatty Acid Profile

The total lipid content was measured by Soxhlet extraction (Velp Scientifica 148, Italy) and calculated on a gravimetric basis. It is expressed in g of lipids/100 g of dry sample [19].

The fatty acid profile was obtained by extracting the lipids and quantified by GC-FID (GC-FID 7890 Agilent Technologies, Santa Clara, CA, USA) [20]. A total of 1 g of sample (accurately weighed) was put into contact with 50 mL of isooctane and 2.5 mL of methanolic solution of KOH, and the resulting solution was homogenized in an ultrasound bath at 80 °C for 20 min. The filtered solution was neutralized with 1 g of NaHSO_4_ × H_2_O. A volume of 1 μL of the neutralized solution was introduced into a FAME gas chromatograph with the flame ionization detector GC-FID 7890A Agilent equipped with db-wax silica capillary column (30 mm × 0.25 mm i.d. × 0.25 μm film thickness). The working conditions were as follows: the injector temperature was set at 250 °C, He gas was used a carrier with a flow rate of 1 mL · min^−1^, and the applied column temperature ranged from 100 °C to 180 °C with a speed of 7 °C/min and a 5 min isothermal regime, followed by an increase from 180 °C to 240 °C at a speed of 10 °C/min and another 10 min isothermal regime. The results are expressed as the fatty acid percentage of the total fatty acids.

#### 2.2.5. Fibres

The crude fibre was quantified according to Commission Regulation (EC) No. 152/2009 [21]. This method involved the acid hydrolysis of 1 g of sample with 150 mL of 0.13 mol/L H_2_SO_4_ at boiling point for 5 min for the extraction of sugars and starch, followed by alkaline hydrolysis with 150 mL of 0.23 mol/L KOH to remove proteins, hemi-cellulose, and lignin. The residue is filtrated, dried, weighed, and ashed at 500 °C for 1 h. A blank test without sample was also conducted. The crude fibre content was calculated according to Equation (2):

Crude fibres (g/100 g) = (m_0_ − m_1_) × 100/m, (2)

where m is the weight of the sample (g), m_0_ is the weight lost after washing (g), and m_1_ is the weight lost after ashing during the blank test (g).

#### 2.2.6. Carbohydrates

The carbohydrate content was calculated by applying Equation (3) [22]:

Carbohydrates (g/100 g) = 100 − (protein + fat + ash + fibre), (3)

#### 2.2.7. Energy

The energy provided by the samples (kcal/100 g) was calculated by considering Annex XIV of Regulation (EU) No 1169/2011 [23], as in Equation (4):

Energy value (kcal/100 g) = 4 × carbohydrate (%) + 4 × protein (%) + 2 × fibre (%) + 9 × fat (%), (4)

### 2.3. Biscuit Making and Sensorial Study Protocols

Four dough formulations were prepared to obtain the corresponding biscuits (Table 1), of which one was reference 0% (R1) and the other three contained 10% (R2), 15% (R3), and 20% (R4) yellow mealworm powder.

The ingredients were mixed for 5 min in a laboratory mixer (Hendi, The Netherlands), and the dough rested for 15 min at 20 °C covered in a sheet of polyethylene to avoid drying. The dough was sheeted (ATLAS, OMG MARCATO, Italy) to 1 cm thickness; then, 6.5 cm diameter round shapes were cut out of the dough sheets, placed on parchment paper in rectangular trays (40 × 30 cm), and baked in a Piron ventilation furnace (Piron SRL, Italy) for 15 min at 180 °C. The biscuits were allowed to cool to room temperature (22 °C). Three batches of each biscuit formulation were prepared, and biscuits from the same formulation were mixed and submitted to the sensory study.

The sensory study of the prepared biscuits was conducted within 5 h of baking at room temperature (22 °C) and in daylight, wherein a group of 20 panellists (10 women and 10 men, aged from 18 to 55 years) who were regular biscuit consumers was employed. The study was approved by the Ethics Committee of the Center for the Scientific Research into Environment, Food, and Health Safety, Technical University of Cluj-Napoca, Romania (458/3 November 2022), and was conducted in according to the rules of the Declaration of Helsinki of 1975 (revised in 2013). The panellists were previously informed of the aim of the study and provided written informed consent. Before the sensorial study, the panellists were trained to develop a consensus on the descriptive vocabulary of the biscuits. Biscuits of each formulation, which were coded and randomly located on the plate, were presented to the panellists. Three scoring sessions were conducted at 15 min intervals, and the panellists rinsed their mouths with water at room temperature between sessions to avoid the carryover effect. The colour, crispness, taste, flavour, cross-sectional structure, tenderness, and overall acceptability were evaluated using a five-point hedonic scale (1—dislike extremely to 5—like extremely). The overall sensory score of each attribute was calculated as an average of the individual scores, and the final results represent the means of two independent experiments. The biscuits containing yellow mealworm powder with the highest overall acceptability attribute scores assigned were selected for further investigations. Yellow-mealworm-powder-free biscuits were also investigated as a reference.

### 2.4. Physical–Chemical Analysis of the Selected Biscuits

#### 2.4.1. Firmness

The firmness, represented by the force required to break the biscuits, was evaluated for at least 10 samples of each type (Wagner FDK10 force dial penetrometer, Wagner Instruments, Riverside, USA), and the average values were reported.

#### 2.4.2. The Diameter (D) and Thickness (T) Ratio

The diameter (D) and thickness (T) of at least 10 samples of each type were measured using callipers, and the spread index (D/T) was calculated based on the average values.

#### 2.4.3. Proximate Analysis and Profiles of Mineral Elements, Fatty Acids, and Amino Acids

The proximate analysis and profiles of the mineral elements, fatty acids, and amino acids in the selected biscuits were measured and expressed as described in Section 2.2.1, Section 2.2.2, Section 2.2.3, Section 2.2.4, Section 2.2.5, Section 2.2.6 and Section 2.2.7.

#### 2.4.4. Determination of 5-Hydroxymethylfurfural

5-Hydroxymethylfurfural (5-hydroxymethyl-2-furaldehyde, HMF), an indicator of changes in food treated with high temperatures, was extracted with Carrez I and Carrez II solutions [24]. The HMF content in the supernatant was measured according to the Winkler method by reading the absorbance at 550 nm [25], and the concentration was calculated based on a calibration curve (R^2^: 0.9991). The results are expressed in mg/Kg.

#### 2.4.5. In Vitro Digestion Studies

To determine the total nutritional value of the selected biscuits, in vitro digestions were carried out in triplicate, wherein the sum of the oral–gastric–intestinal stages was considered and the INFOGEST protocol [26] detailed in the Supplementary Material was followed. Several parameters were considered, as follows: the bioaccessibility of protein and fat, glycaemic index, and the fractions of rapidly digestible starch (RDS) and slowly digestible starch (SDS).

Samples were collected at the end of the digestion stage, shock-treated in an ice bath for 5 min to ensure enzyme inactivation, and centrifuged at 6000× *g* for 30 min to separate the supernatant. Accurately measured 15 mL aliquots of the supernatant were used to measure the total protein content via Kjeldahl assay (as described in Section 2.2.3) and the total fat content via Soxhlet extraction (as described in Section 2.2.4). Blank digestions were run solely using simulated bodily fluids, and the protein and fat concentrations were measured as they were in biscuit sample digestion. The final results were calculated as the difference between the protein and fat amounts in the sample and the blank digestion fluids. The bioaccessible fractions of protein and fat were calculated as follows:

Bioaccessible fraction (%) = (M_S_ − M_Blank_) × 100/M_B_, (5)

where M_S_ and M_Blank_ are the amounts of protein or fat in the supernatant of the digested biscuit and blank digestion fluids, respectively (mg/100 g), and M_B_ is the total amount of protein or fat in the undigested biscuit sample (mg/100 g).

The glycaemic index of the biscuits was calculated according to the method proposed by Yusufoğlu et al. [27], which was adapted to our work. Samples of digesta were collected every 5 min in the first 30 min and every 10 min from 30 to 180 min, placed in an ice bath, and centrifuged at 6000× *g* for 30 min to separate the supernatant. The digestion procedure was started with individual tubes for each time point to ensure higher reproducibility of the results. The amount of glucose was measured in 10 μL of supernatant by adding 1 mL of commercially available glucose oxidase/peroxidase D-glucose assay kit (Biosystems, Spain), which measures the decomposition of glucose to gluconate under the action of glucose oxidase. The resulting H_2_O_2_ reacted with 4-aminoantipirin and phenol to form a red compound. The absorbance of this compound was read by a spectrophotometer at 500 nm against a blank after incubation for 10 min at 37 °C (BTS-350 analyser, Biosystems, Spain). A blank digestion procedure was also conducted following the same protocol. The glucose concentrations (mg/g sample) were calculated at each sampling point, and the final result was quantified as the difference between the glucose amounts in the sample and the blank digestion. The amount of glucose was plotted against the sampling times (hydrolysed curve), and the area under the hydrolysed curve (AUHC_B_) was calculated using Excel. In vitro digestion was conducted using 0.1 mL of standard D-glucose instead of the biscuit samples, and a hydrolysed curve and the area under the hydrolysed curve (AUHD_S_) were determined following the same procedure.

The hydrolysis index (HI) of the glucose in the biscuits was calculated according to Equation (6) [28]:

HI = AUHC_S_ × 100/AUHC_B_, (6)

where AUHC_B_ and AUHC_S_ represent the area under the hydrolysed curve for biscuits and D-glucose samples, respectively.

The estimated glycaemic index (eGI) of the biscuits was calculated using Equation (7) [29]:

eGI = 0.7[39.71 + (0.559 · HI)].(7)

The starch content of the biscuit samples was measured according to the AACC 76–13 method [30]. It is based on the hydrolysis of starch into maltodextrins by α-amylase, followed by their hydrolysis to D-glucose in the presence of amyloglucosidase. D-Glucose is oxidised to D-gluconate with the release of H_2_O_2_, which can be quantitatively measured at 510 nm in a colourimetric reaction employing peroxidase, and the production of a quinoneimine. Depending on its hydrolysis rate in the small intestine under the action of α-amylase, the starch is classified into three fractions: (i) rapidly digestible starch (RDS), which hydrolyses within the first 20 min, resulting in the rapid release of glucose from the small intestine into the bloodstream; (ii) slowly digestible starch (SDS), which hydrolyses between 20 and 120 min, resulting in prolonged glucose release into the bloodstream; and (iii) resistant starch (RS), which remains undigested after 120 min [31], and is degraded through glycolysis by microorganisms in the colon. The fractions of RDS (%) and SDS (%) in the samples were calculated according to Equations (8) and (9) [5]:

RDS (%) = (G_20_ − G_0_) × 0.9 × 100/TS, (8)

SDS (%) = (G_120_ − G_20_) × 0.9 × 100/TS, (9)

where G_0_ is the free glucose content of the sample (mg/g sample), G_20_ is the glucose released within 20 min (mg/g sample), TS is the total content of starch (mg/g sample), G_120_ is the glucose released within 120 min (mg/g sample), and 0.9 is the conversion factor of glucose into starch.

The digestion graph was plotted as the ratio of total starch digested against the digestion time. Experimental results were fit to starch digestion kinetic models, for which it was considered that the hydrolysis of SDS occurs at a lower rate than RDS. Digestion can occur either in parallel or sequentially and conforms to the first-order kinetic model. Two kinetic models were considered to quantify the dissimilarities between the digestion of RDS and SDS fractions [32]:

(i) the logarithm of slope model (LOS) distinguished whether there were multiple digestion fractions with different digestion rates by using a variation profile of starch concentration during digestion, as described by Equation (10):

(10) $$\frac{\mathrm{dc}}{\mathrm{dt}}=-K\cdot C,$$

where c is the amount of starch digested at time t (%), t is the digestion time (min), K is the first-order kinetic coefficient (min^−1^), and C_0_ is the starch concentration at the initial time (C_0_ is the starch concentration at the initial time (%)).

By integrating Equation (10) between the limits C_0_ and C_∞_, the following relation was defined:

C(t) = C_0_ + (C_∞_ − C_0_)·(1 − e^−Kt^), (11)

where C(t) is the ratio of digested starch at time t (%), C_0_ is the digestion ratio of starch at the beginning of the reaction (%), and C_∞_ is the estimated maximum starch digestion ratio under the infinite extension of reaction time (%).

By taking the logarithm of Equation (11), the LOS equation is obtained:

(12) $$\ln\frac{\mathrm{dC}}{\mathrm{dt}}=\ln[K\cdot {(C}_{\infty }-C_{0})]-K\cdot t.$$

The LOS plotted versus digestion time results in two distinct lines with different slopes attributed to the digestion of the RDS and SDS fractions. Their intersection allows for the estimation of the starting time for the digestion of the SDS fraction (ts).

(ii) The parallel sequence model (CPS) [32,33] considers the digestion of both starch fractions (RDS and SDS), and the amount of digested starch at each moment of the process can be quantified as the sum of the amounts digested from the two fractions, as described by Equation (13):

(13) $$C(t)=C_{\mathrm{RSD}}+C_{\mathrm{SDS}}=C_{\mathrm{RDS}0\;}+{(C}_{\mathrm{RDS}\infty }-\;C_{0})(1-e^{-K_{\mathrm{RDS}}\cdot t})+{\mathrm{IF}[t}_{s}\geq {t,C}_{\mathrm{SDS}0}+{(C}_{\mathrm{SDS}\infty }-C_{0})(1-e^{-K_{\mathrm{SDS}}\cdot (t-t_{s})}),\;\;0]$$

Considering that there is no digested starch initially, Equation (13) is simplified to the form:

(14) $$C(t)=(C_{RDS\infty }-C_{0})(1-e^{-K_{RDS}\cdot t})+IF[t_{s}\geq t,(C_{SDS\infty }-C_{0})(1-e^{-K_{SDS}\cdot (t-t_{s})}),0]$$

where C_RDS∞_ and C_SDS∞_ are the ratios of digested RDS and SDS at infinite reaction time (%); K_RDS_ and K_SDS_ are the RDS and SDS digestion rate coefficients, respectively (min^−1^); and ts is the starting time for the digestion of the SDS fraction (min).

The parameters of the kinetic models, C_RDS∞_, C_SDS∞_, K_RDS_, K_SDS_, and ts, were quantified using the non-linear least squares method (NLLS) by minimising the error sum of squares in Free Pascal (General Public Licence).

Details about the physical–chemical analysis protocols are provided in the Supplementary Material.

### 2.5. Statistical Analysis

At least three independent measurements were performed in each experiment. The results are presented as mean value ± standard deviation (SD). One-way ANOVA (IBM SPSS Statistics 24) was used to assess the difference between the means, and the probability value of *p* < 0.05 was considered statistically significant. The linear regression applied in the LOS model was conducted using Statistica 7.0 (StatSoft. Inc., USA).

## 3. Results and Discussion

### 3.1. Proximate Compositions of White Wheat Flour and Yellow Mealworm Powder

The proximate compositions (expressed on a dry basis) of white wheat flour (WWF) and yellow mealworm powder (YMP) are shown in Table 2. Significant differences (*p* < 0.05) were found between the investigated samples. The main components of WWF are carbohydrates (88.04 g/100 g), of which starch is the largest proportion [34]. The concentration of total proteins in WWF was 9.87 g/100 g. Thus, this flour is considered of superior cooking quality. Our results were close to those reported by Nisar et al. [35]. Unlike WWF, the main compounds in the YMP were proteins (48.21 g/100 g) and lipids (34.21 g/100 g), and this result is in agreement with the values of Gonzales et al. [8]. The removal of fibre during the milling of the wheat grain explains the low quantity of fibre in WWF (0.51 g/100 g). The fibre in the insect powders consists mainly of sclerotised proteins and chitin, i.e., exoskeletal components [36].

As shown in Table 2, the amount of ash in YMP is 7.40-fold higher than that in WWF due to the higher concentrations of individual mineral elements in YMP compared to WWF.

During the milling of wheat grains, large amounts of minerals are removed with the bran. Oghbaei and Prakash [37] have reported a 90% decrease in Mn; 80% decreases in Mg, K, and Cu; a 33% decrease in Ca; and an 85% decrease in Zn in hard white wheat flour compared to hard whole wheat flour. In addition, Fe, Zn, and P were reduced 1.65-, 1.58-, and 2.34-fold, respectively [38]. In the WWF, the concentration of Na was the largest (180.02 mg/100 g), followed by K (123.45 mg/100 g) and P (112.20 mg/100 g). A significant amount of phosphorus in plants is found as phytate and hence is unavailable for digestion [36]. Like other plants, wheat grains contain non-heme iron, mostly in the form of Fe^3+^ ions, whose absorption into blood requires conversion to Fe^2+^ ions, which are more easily absorbable. Potassium predominated in the YMP; it was found at a higher concentration (865.03 mg/100 g) than in WWF. Magnesium was the second highest mineral element in YMP (332.16 mg/100 g), followed by magnesium (332.16 mg/100 g) and sodium (265.12 mg/100 g). The lack of a mineralised skeleton explains the low level of Ca in YMP (215.15 mg/100 g), which was below the value of 0.3% found in the literature [36]. The other investigated mineral elements had higher concentrations in YMP than in WWF. YMP is a 13.75-fold richer source of iron compared to WWF. The use of YMP is thus a promising approach for improving iron status in humans. In insects, iron is primarily present as the non-heme molecules of ferritin and holoferritin, which can bind thousands of Fe ions, typically in the ferrous (Fe^2+^) state [39]. The P, Cu, Zn, Mn, and Li concentrations were 1.98-, 158.73-, 120.04-, 16.23-, and 2.71-fold higher in YMP than in WWF. Unlike WWF, the phosphorus in insects is likely bioavailable [40].

A significant difference was observed between the fatty acid profiles of WWF and YMP (Table 3).

In WWF, the fatty acid profile was dominated by linoleic acid C18:2, *n*-6 (61.47%); followed by oleic acid C18:1, *n*-9 (16.65%); and palmitic acid C16:0 (14.24%). Lower amounts were found for linolenic C18:3, *n*-3 (2.67%); stearic C18:0 (1.92%); gondoic C20:1; *n*-9 (0.75%); behenic C22:0 (0.62%); palmitoleic C16:1, *n*-7 (0.50%); capric C10:0 (0.42%); caprylic C8:0 (0.35%); pentadecanoic C15:0 (0.21%); and myristic C14:0 (0.20%) acids. The predominance of linoleic acid in WWF was also reported by Roncolini et al. [41], namely, a concentration of 60.72%. Linoleic acid was also the most abundant in YMP but was present in a smaller amount (38.90%) compared with WWF. However, oleic and palmitic acids were more abundant in YMP, accounting for 28.13% and 15.78%. Compared to WWF, larger percentages of C14:0; C18:0; C16:1, *n*-7; and C18:3, *n*-3 were found in YMP. Arachidic C20:0 and myristoleic C14:1, *n*-5 acids not detected in WWF were found in YMP, while caprylic C8:0, capric C10:0, and behenic C22:0 acids were not detected in YMP. The PUFA/SFA ratio in WWF is 1.49-fold higher than in YMP. Our ratio of 2.41 for YMP is higher than the 0.59 ratio previously reported by Kowalski et al. [42]. Regarding the *n-6/n-3* ratio, the value of 3.81 for YMP is lower than the value of 23.02 obtained for WWF and close to the recommended range of 1:1 to 1:5 [41]. A low *n-6/n-3* ratio in YMP suggests a higher amount of *n*-3 fatty acids. The water-soluble amino acid profiles are displayed in Table 4. WWF showed a higher amount of nonessential amino acids (60.55%), represented by glutamic acid (28.01%) and proline (14.06%), and a lower amount of essential amino acids (39.45%), including leucine (15.18%), isoleucine (8.04%), and valine (7.34%). Enrichment in essential amino acids of up to 56.48% was observed in YMP, including 18.53% leucine, 9.11% valine, 0.45% methionine, 6.19% phenylalanine, 0.21% lysine, and 11.92% tyrosine. It should be noted that the developmental stages of insects affect their composition.

### 3.2. Sensory Study and Selection of the Biscuits for Further Investigations

**Figure 1:**
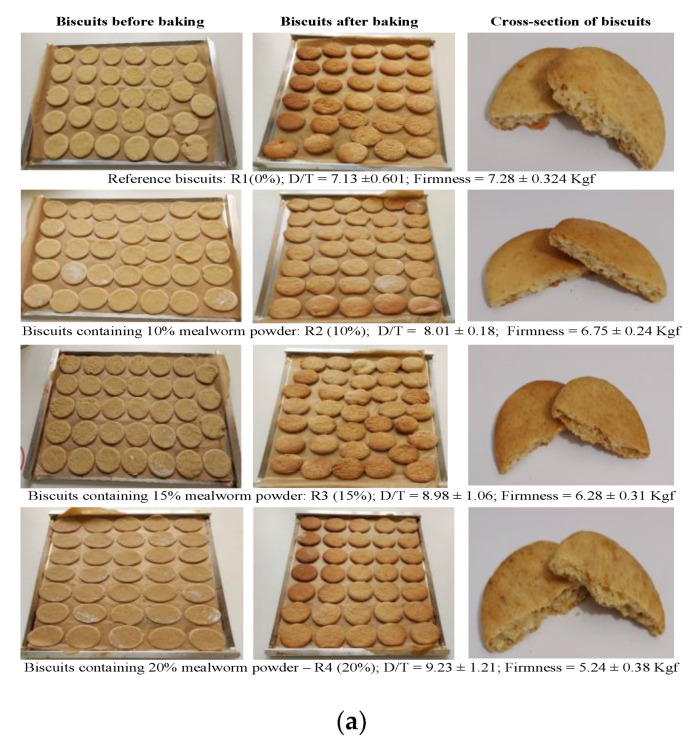

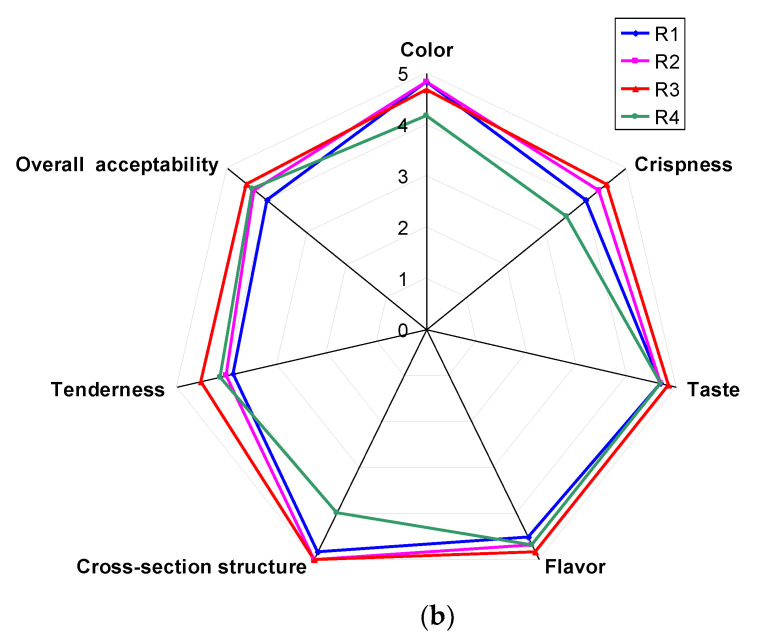
Appearance of prepared biscuits (**a**) and the results of their sensorial analysis (**b**): R1—reference biscuits; R2—biscuits with 10% yellow mealworm powder; R3—biscuits with 15% yellow mealworm powder; R4—biscuits with 20% yellow mealworm powder.

The evaluation of the prepared biscuits (Figure 1a,b) shows changes in their sensory attributes and the perceptions of consumers with the addition of mealworm powder. The R1 and R2 biscuits received the highest scores for their light brown colour. The increase in the ratio of YMP resulted in a less-accepted intense brown colour, which was produced due to the larger amounts of mellanoidins that formed via Maillard reactions. The mechanical properties of the biscuits were influenced by the mealworm powder’s addition, with fat being the main ingredient responsible for the tenderness and texture of biscuits. The reference biscuits (R1) presented a higher breaking force of 7.28 Kgf (Figure 1a), which was accounted for by the panellist with the lowest score concerning the tenderness attribute (3.86). The higher amount of fat yielded by YMP resulted in softer mealworm powder-biscuits. Their breaking points decreased in the range of 6.75–5.24 Kgf with the increase in the YMP ratio, and they were better accepted by the consumers, who gave them scores of 4 for R2, 4.5 for R3, and 4.12 for R4. The crispiness was also affected by the inclusion of mealworm powder due to the high amounts of fatty acids and proteins in YMP. These components diluted the gluten, altered the cohesiveness and extensibility of the gluten network, increased the diameters of the biscuits, and resulted in higher spread (D/T) ratios. The R3 biscuits were the best-liked biscuit in terms of crispness, while the R4 biscuits were the most disliked. The panellists highly appreciated the taste and flavour of the R3 biscuits and assigned them the highest scores of all the biscuits (4.83). The substitution of WWF with YMP can result in an unpleasant aroma due to this ingredient’s high content of fatty acids that release an off-flavour smell during baking. However, this was not the case in our study, as the particular aroma of the yellow mealworm powder biscuits was appreciated, with higher scores of 4.67, 4.83, and 4.67 compared to that assigned to the R1 biscuits (4.5). Larger and irregular pores that were unevenly distributed inside the R4 biscuits were assigned the lowest score (3.98) with respect to the cross-section attribute, while the highest score was obtained by the R1 and R3 biscuits. With regard to the overall assessment, the same trend was expressed concerning R4, R1, and R3, with assigned scores of 4, 4.5, and 4.5, respectively. With six attributes rated with the highest scores out of a total of seven, the 15% substitution of WWF with YMP powder seems to be a threshold for the fortification of this type of biscuit with mealworm powder. As a result of the sensory study, the biscuits enriched with 15% yellow mealworm powder R3 (Figure 1a,b) were selected for further investigation, as they had the highest overall acceptability attribute. Biscuits made entirely from white wheat flour R1 were also investigated as a reference.

### 3.3. Characterisation of Selected Biscuits

#### 3.3.1. Proximate Composition of Selected Biscuits

Table 2 reports the proximate analysis of the investigated biscuits. The addition of YMP resulted in increased amounts of proteins, lipids, and fibre in the R3 biscuits, which occurred in parallel with a 1.21-fold reduction in carbohydrates compared to the reference biscuits R1. No significant differences in the energy contents of the two types of biscuits were observed.

As can be observed in Table 2, the 5-HMF levels were unexpectedly low in both types of biscuits given their high reducing sugar and amino acid content, which are 5-HMF-generating substrates. The low temperature and short baking time can be connected with the low HMF levels [43]. Another factor favouring the low level of 5-HMF is the nature of the leavening agent. Sodium bicarbonate prevents the decomposition of the sugars by increasing the pH inside the dough. Subsequently, the production of 5-HMF is avoided. Gökmen et al. [44] have reported a 20-fold decrease in 5-HMF concentration in cookies made with saccharose and a 2-fold decrease in cookies with glucose when ammonium bicarbonate was replaced with sodium bicarbonate. The addition of YMP contributed to a 1.49-fold reduction in the amount of 5-HMF generated in the R3 biscuits; even the protein content in R3 was 1.08-fold higher than in R1. The quantity of carbohydrates was 1.21-fold lower in R3 compared to R1, accompanied by a lower humidity (5.02%), compared to 7.66%. These factors favoured the generation of less 5-HMF in R3.

Regarding the mineral elements, adding 15% YMP enriched the biscuits in terms of potassium, calcium, magnesium, copper, zinc, manganese, and iron content (Table 2). The elevated concentrations of P in both types of biscuits were due to the incorporation of soy milk and vanilla sugar. Although YMP is a richer source of P than WWF, its incorporation at 15% into the dough did not lead to yellow mealworm biscuits with significantly increased amounts of P compared to the reference biscuits. The Ca:P molar ratios of 0.09:1 in the reference biscuits and 0.1:1 in the yellow mealworm biscuits did not fall within the reference molar range from 1.4:1 to 1.9:1 recommended by the EFSA [45], suggesting that the absorption of Ca was difficult. Likewise, no significant differences were observed between the Li concentrations of the two biscuit samples.

Table 3 displays the fatty acid profiles of the investigated biscuits. Linoleic acid was the most abundant fatty acid in the reference (60.11%) and yellow mealworm (58.72%) biscuits due to its prevalence in white wheat flour. PUFAs and MUFAs accounted for 63.71% and 20.24% in R1 and 62.76% and 20.94% in R3, but no significant differences (*p* < 0.05) were detected between values. The nutritional quality of the lipids in the investigated biscuits was assessed by calculating several ratios and indexes according to the equations proposed by Roncolini et al. [41]. The recommended *n*-6/*n*-3 ratio of 1:1 to 1:5 was not found in the investigated biscuits. The yellow mealworm and reference biscuits had similar values of the atherogenic (AI) and thrombogenic (TI) indices. According to Roncolini et al. [41], AI and TI values higher than 1.00 and PUFA/SFA ratios greater than 0.4 are considered appropriate for a healthy dietary oil/fat intake.

The water-soluble amino acid profiles (Table 4) indicated a higher level of essential amino acids EAAs of 50.31% in the R3 biscuits compared to 45.07% in R1 biscuits. Regarding EAAs, a larger percentage of valine was found in R3 than in R1, while the nonessential amino acids (NEAAs) proline, glutamic acid, and asparagine prevailed in R1.

#### 3.3.2. In Vitro Digestion of Protein and Fat in Selected Biscuits

The protein digestion process begins in the stomach with pepsin action, continues in the intestine with trypsin and chymotrypsin action, and is completed on the intestinal surface by protease action [46]. In our study, the highest protein digestibility value (Table 2) was found in the yellow mealworm biscuits, specifically, 85.14%, which is 1.12-fold higher than the reference biscuits (76.31). Accardo et al. [47] have found that the solubilisation of proteins from lesser mealworm (*Alphitobius diaperinus*) powder was 76.5% in samples subjected to oral–gastric–duodenal digestion. Several factors could be considered to be responsible for the more efficient solubilisation of proteins from yellow mealworm biscuits. The electrophoretic profile of wheat flour in the study of Kowalski et al. [42] indicates gliadins within the molecular range 30–50 kDa are the most visible protein band, accompanied by low-molecular-weight subunits of glutenins. In the case of the yellow mealworm powder investigated in the same study, most proteins had lower molecular weights, between 10 and 20 kDa, making them more susceptible to digestion. Replacing WWF with YMP diluted the gluten network of the biscuits and resulted in a less dense and viscous structure, which favoured access by digestive juices and proteases to the digesta, and, consequently, increased the level of protein digestibility. The high temperature associated with baking could denature the protein-unfolding polypeptide chains, thereby increasing the access of protease and inactivating the antinutritional compounds that may inhibit the action of enzymes [48]. Roasting, frying, boiling, steaming, and drying are among the commonly used thermal methods for processing insects. The literature has reported contradictory effects of these processes on the digestibility of proteins. Capparos Megido et al. [49] reported a significant increase in protein digestibility for boiled and oven-cooked mealworms. According to the work of Mancini et al. [48], deep frying showed the highest reduction in simulated protein gastric digestibility, followed by oven cooking for 10 min, pan frying, and boiling, with minor differences compared to oven cooking for 30 min, microwaving, and steaming. Table 2 shows no significant differences in terms of lipid bioaccessibility in the R3 and R biscuits. Similar behaviour has been observed in the study of Bas and El [13], who reported no significant difference between lipid digestibility in biscuits made with wheat flour (57.74%) and biscuits made by replacing 20% of wheat flour with cricket powder (55.56%). The degree of lipolysis is strongly affected by the rheology of the food matrix, the distribution of oil droplets, and hydrolysis kinetics. Bas and El [13] have shown that the lipid hydrolysis in biscuits enriched with 20% cricket powder was rapid in the first 30 min and then gradually slowed because of the accumulation of lipolytic products at the interface, which prevents enzymes from accessing the triglyceride. Accardo et al. [47] have reported the poorer solubility of lipids (23%) from lesser mealworm powder during oral–gastric–duodenal digestion.

#### 3.3.3. Kinetics of Starch Hydrolysis and Estimated Glycaemic Index (eGI) Values

**Figure 2:**
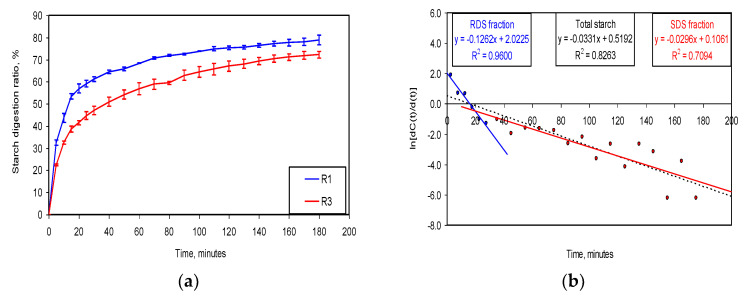

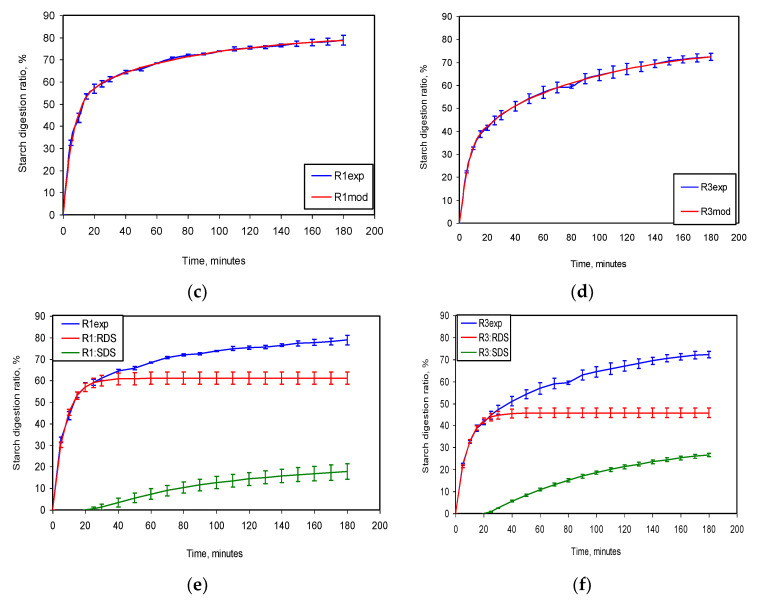
Application of LOS and NLLS kinetic models to the in vitro starch digestion results: RDS—rapidly digestible starch; SDS—slowly digestible starch; Total starch—the total amount of starch; R1exp and R3exp—experimental profiles for starch digestion ratio for R1 and R3 biscuits; R1mod and R3mod—modelled profiles for starch digestion ratio for R1 and R3 biscuits. (**a**)—Experimental starch digestion profiles in reference biscuits (R1) and 15% yellow mealworm-biscuits (R3); (**b**)—LOS method fit to the experimental results from the first replica of the starch digestion experiments in R1 biscuits, where line represents rapidly digestible starch fraction (RDS), red line represents slowly digestible starch fraction (SDS), and black line represents total starch digestion (overall fit curve); (**c**)—comparative analysis between experimental and NLLS-modelled starch digestion profiles in reference biscuits (R1); (**d**)—comparative analysis between experimental and NLLS-modelled starch digestion profiles in 15% yellow mealworm biscuits (R3); (**e**)—NLLS-modelled discrimination of total starch digestion profile in R1 biscuits between digestion profiles of RDS and SDS fractions; (**f**)—NLLS-modelled discrimination of total starch digestion profile in R3 biscuits between digestion profiles of RDS and SDS fractions.

As Figure 2a shows, the experimental results regarding starch digestion display a typical exponential trend for both types of biscuits. The values concerning the relative standard deviation (RSD, %) ranged between 0% and 4.58%. The digestion of starch in the first 20 min, assumed to be the digestion of the RDS fraction, occurred at the highest level in the reference biscuits (56.92%) and a lower level in the yellow mealworm biscuits (41.47%). In both types of biscuits, the starch digestion after 20 min slowed until it reached a plateau.

The LOS and NLLS kinetic models were used to characterise starch digestion.

In the *LOS method*, for each biscuit type and each triplicate, the variation profiles for $\ln\frac{\mathrm{dC}}{\mathrm{dt}}$ during digestion were recorded. For each case, linear regression models corresponding to the evolution of the total starch, RDS, and SDS fractions were evaluated. Figure 2b provides an example of the application of the LOS method for the first replicate of the digestion experiments for the R1 biscuits. The presence of two fractions in the starch digestions was assumed based on the different slopes of the profiles [50]. The analysis of the experimental data covers four aspects: (i) the characterisation of the linear regression models by generating the coefficients of the intercept (a) and slope (b) of the models, and assessing the accuracy of these models using the correlation coefficients R, R^2^, Adj R^2^ (adjusted R), and SEE (standard errors of estimate); the absolute values of the slopes corresponding to the rate coefficients in the starch digestion kinetics equations; (ii) ANOVA analysis of the linear regression models, which establishes the level of statistical significance; (iii) statistical analysis of the coefficients of the linear regression models; and (iv) the coordinates of the intersection point of the lines corresponding to the digestion of the RDS (blue line in Figure 2b) and SDS (red line in Figure 2b) fractions assimilated at the initiation time of the digestion of the SDS fraction. Table 5 summarises the data extracted from the example displayed in Figure 2b. The LOS method is associated with large errors due to the numerical derivation of the concentration variation profiles as a function of time.

The SEE values across the various linear regression models ranged between 0.2533 and 0.9934, while the corresponding Adj R^2^ values ranged between 0.1851 and 0.9499. However, ANOVA analysis revealed 17 (of 18) statistically significant linear models, and a further inspection of the slopes revealed that 11 (of 18) were statistically significant (*p* < 0.05). Table 6 shows the average rate coefficients (K_RDS_ and K_SDS_) and ts associated with the digestion of the RDS and SDS fractions fitted to the LOS model.

The application of the *NLLS model* to describe the kinetics of starch digestion in the investigated biscuits resulted in SEE values ranging between 1.4526 and 21.3889, corresponding to average percentage errors of 0.4598–1.4021%These values were calculated as the average of the absolute individual errors between the experimental and modelled degradation ratios corresponding to each moment of digestion. By applying the minimisation of quadratic errors (SS), the individual values for the independent variables C_RDS∞_, C_SDS∞_, K_RDS_, K_SDS_, and ts were calculated, and their average values are listed in Table 6.

Figure 2c,d compare the experimental and modelled starch digestion profiles, while in Figure 2e,f, the total starch digestion profile is differentiated in the digestion profiles of RDS and SDS fractions, respectively.

The analysis of the values of the rate coefficients (K_RDS_, K_SDS_) and starting times for the digestion of the SDS fraction (ts) determined using the LOS and NLLS methods (Table 6) revealed the following:

(1) For both types of biscuits, lower values for the rate coefficients associated with the RDS fractions were obtained using the LOS model compared to the NLLS model.

(2) Regardless of the mathematical model, no significant differences were observed between the rate coefficients associated with the digestion of the SDS fraction.

(3) In both kinetic models, the rate coefficients assigned to the RDS fraction were lower in the R3 biscuits compared to R1, but only the LOS model showed a statistically significant difference (*p* < 0.05) between them. No significant differences occurred between the rate coefficients regarding the digestion of the SDS fraction.

(4) Statistically significant differences were revealed between C_RDS∞_ and C_SDS∞_ in the reference and yellow mealworm biscuits, respectively. The maximal digestion range for the RDS fractions (C_RDS∞_) was 1.33-fold reduced in R3 compared to R1. For the SDS fraction, C_SDS∞_ increased 1.53-fold. This indicated that adding yellow mealworm powder resulted in a larger amount of SDS and, subsequently, a decreased RDS fraction.

(5) The LOS and NLLS models are reliable and accurately predict the in vitro time when the transition from fast to slow starch digestion occurs. As shown in Table 5, there are no significant differences between the ts values for the R1 and R3 biscuits. This result confirms the absence of statistical differences between the rate coefficients for the digestion of the SDS fraction, suggesting that the SDS resulted from the addition of the yellow mealworm powder; it did not hydrolyse faster than the SDS initially present in white wheat flour. However, the ts values resulting from the application of the LOS and NLLS models were higher than the ts value (20 min) resulting from the general digestion trends of the biscuits (Figure 2a), and this implies that starch digestion follows a pattern between the sequential and parallel modes. The digestion of the RDS and SDS fractions did not start simultaneously but overlapped. A similar starch digestion model has been documented in sprouted oat flour-based bread [50], while a sequential mode has been reported for starch from barley-based noodles [51].

After 180 min, the highest starch digestion ratio (78.79%) was achieved in R1, while that of 72.26% was achieved in R3 (Table 2). This indicated a 1.09-fold reduction in the starch digestibility of R3 and resulted in a lower glycaemic response. The estimated glycaemic index in the R3 biscuits (79.56%) was 1.13-fold lower than in the R1 biscuits (90.14%). Our values are close to the glycaemic index values of 81.16% and 92.22% that have been reported by Zielinska et al. [9] for muffins enriched with 10% yellow mealworm powder and reference muffins, respectively.

Different factors, such as the starch quantity and granular characteristics (morphology, amylose to amylopectin ratio, and degree of branching), the physical state (integrity of cell walls), and the non-starch constituents of the food matrix (proteins, lipids, and fibres), play significant roles in the digestibility of starch [52]. The incorporation of YMP into the dough significantly (*p* < 0.05) increased the protein, fat, and fibre content of the R3 biscuits, which occurred in parallel with the reduced starch content, compared to the R1 biscuits.

Starch gelatinisation during baking destroys the initial organised structure of the starch and makes it easy to digest, thus rapidly increasing the level of glucose and insulin in the blood. YMP absorbs more water than WWF due to its higher protein content, which reduces the water available for starch hydration, lowers its gelatinisation degree, and decelerates the digestion rate. Polyphenols, chitin, tannins, alkaloids, and saponins that have been reported in YMP [53] act effectively as inhibitors [54], restrain the amylase action leading to a reduction in starch hydrolysis, and hence lower the blood glucose level. Proteins and fats, which naturally occur in the food matrix, may inhibit starch hydrolysis due to starch–lipid–protein complexes.

The behaviour of amylose, The linear and branched chains are associated to single-helical complexes with lipids and not to amylose has been documented [55]. The thermal treatment of such complexes above their dissociation temperature results in the rearrangement of their structure from amorphous to lamellar crystallites. Even in small amounts, proteins have also been reported to entrap the starch by forming a network around the starch molecule [56]. Such a compact network structure is characterised by the enhanced entrapment of starch and offers improved steric hindrance [57], acts as a barrier against starch digestion by limiting the contact surface area between the starch and amylase, and affects the binding of the enzyme to the substrate. The increased fibre intake decreases the digestion of starch in the small intestine due to its starch isolation action [58] and amylase inhibition. The factors mentioned above may account for the retardation of the starch digestion rate due to reduced sensitivity to the amylase action, thus reducing the RDS fraction, increasing the SDS fraction, and decreasing the glycaemic response of the R3 biscuits compared to the R1 biscuits.

## 4. Conclusions

This study was conducted to identify the potential of yellow mealworm (*Tenebrio molitor*) powder to enhance the nutritional value of biscuits. The proximate analysis revealed higher quantities of proteins, total lipids, total mineral elements, and crude fibre in the yellow mealworm powder than in the white wheat flour, and a lower quantity of carbohydrates. The yellow mealworm powder is a richer source of Na, K, Ca, Mg, P, Cu, Zn, Mn, Fe, and Li than white wheat flour. White wheat flour presented a higher amount of nonessential amino acids (60.55%), while essential amino acids prevailed in the yellow mealworm powder (56.48%). Monounsaturated fatty acids accounted for a 30.49% share in the yellow mealworm powder, and polyunsaturated fatty acids were at the highest level in the white wheat flour (64.14%). Compared with the biscuits made of 100% white wheat flour, the addition of 15% yellow mealworm powder enhanced the amounts of protein, fat, fibre, and minerals in the biscuits while reducing the carbohydrate and 5-HMF content. Regarding the essential amino acids, valine was present in a higher concentration in the yellow mealworm biscuits (5.97%), while glycine (3.76%) and arginine (6.09%) were the most representative non-essential amino acids. No significantly significant differences were observed between the concentrations of saturated fatty acids, monounsaturated fatty acids, and polyunsaturated fatty acids in the white wheat biscuits and yellow mealworm biscuits. A 1.12-fold higher level of bioaccessibility of the protein fraction was observed during the digestion of 15% yellow mealworm biscuits. No significant difference was observed regarding the bioaccessible fractions of fat in the yellow mealworm and reference biscuits. Applying the LOS and NLLS kinetics models to the experimental results regarding in vitro digestion showed the presence of both rapidly and slowly digested starch fractions. The kinetics models did not indicate significant changes in the rate coefficients of the slowly digestible starch fraction in the reference and yellow mealworm biscuits but revealed increased slowly digestible and decreased rapidly digestible starch fractions in the 15% yellow mealworm biscuits. These changes resulted in a 1.09-fold decrease in the total starch digestibility and a 1.13-fold reduction in the glycaemic response of the 15% yellow mealworm biscuits compared to the reference biscuits. Due to their enhanced nutritional profile, higher fraction of bioaccessible protein, and lower glycaemic index, the biscuits enriched with 15% yellow mealworm powder can be considered a good alternative to traditional biscuits.

## Figures and Tables

**Table 1:** Dough and corresponding biscuits’ formulation and coding.

Ingredients	Dough and Corresponding Biscuits’ Formulation and Coding (Ratio of Yellow Mealworm Powder *)			
	R1 (0%)	R2 (10%)	R3 (15%)	R4 (20%)
White wheat flour, g	200	180	170	160
Yellow mealworm powder, g	0	20	30	40
Sodium bicarbonate, g	5	5	5	5
Sunflower oil, mL	50	50	50	50
Soy milk, mL	85	85	85	85
Honey, g	50	50	50	50
Vanilla sugar, g	8	8	8	8
Salt, g	1	1	1	1

* Yellow mealworm powder ratio is reported as the total amount of white wheat flour + yellow mealworm powder.

**Table 2:** Proximate analysis of white wheat flour, yellow mealworm powder, and selected biscuits.

Parameter		Raw Materials		Selected Biscuits	
		White Wheat Flour WWF	Yellow Mealworm Powder YMP	Reference Biscuits (100% White Wheat Flour) 0% (R1)	Biscuits Containing 15% Yellow Mealworm Powder 15% (R3)
Dry matter g/100 g		90.27 ± 2.62 (a)	93.52 ± 3.11 (a)	92.34 ± 1.56 (b)	94.98 ± 0.42 (a)
Protein, g/100 g		9.87 ± 0.29 (b)	48.21 ± 2.20 (b)	29.11 ± 0.46 (b)	31.33 ± 0.53 (a)
Total lipids, g/100 g		0.90 ± 0.06 (b)	34.21 ± 0.97 (a)	46.43 ± 1.10 (a)	47.81 ± 1.55 (a)
Ash, g/100 g		0.68 ± 0.03 (b)	5.03 ± 0.26 (a)	1.42 ± 0.09 (b)	1.56 ± 0.04 (a)
Crude fibre, g/100 g		0.51 ± 0.02 (b)	4.01 ± 0.13 (a)	1.36 ± 0.12 (b)	1.42 ± 0.05 (a)
Carbohydrates, g/100 g		88.04 ± 0.52 (a)	8.54 ± 0.32 (b)	21.68 ± 1.12 (a)	17.88 ± 1.71 (b)
Energy, kcal/100 g		400.76 ± 10.30 (b)	542.91 ± 7.54 (a)	621.95 ± 5.72 (a)	629.97 ± 4.36 (a)
5-HMF, mg/Kg		-	-	6.21 ± 0.08 (a)	4.17 ± 0.12 (b)
Bioaccessible fraction of total protein (%)		-	-	76.31 ± 3.29 (b)	85.14 ± 4.35 (a)
Bioaccessible fraction of total fat (%)		-	-	54.07 ± 3.34 (a)	56.38 ± 4.02 (a)
Estimated Glycaemic Index, %		-	-	90.14 ± 3.14 (a)	79.56 ± 3.24 (b)
Mineral elements, mg/100 g	Na	180.02 ± 2.11 (b)	265.12 ± 3.45 (a)	381.05 ± 9.70 (b)	389.04 ± 4.87 (a)
	K	123.45 ± 2.32 (b)	865.03 ± 7.12 (a)	386.48 ± 3.32 (b)	413.84 ± 3.11 (a)
	Ca	43.17 ± 1.67 (b)	215.15 ± 4.83 (a)	98.29 ± 1.67 (b)	104.58 ± 1.86 (a)
	Mg	32.45 ± 2.13 (b)	332.16 ± 3.13 (a)	81.33 ± 1.76 (b)	98.12 ± 2.11 (a)
	P	112.20 ± 3.02 (b)	222.45 ± 3.20 (a)	779.44 ± 2.45 (a)	780.11 ± 2.33 (a)
	Cu	0.11 ± 0.01 (b)	17.46 ± 0.45 (a)	1.77 ± 0.012 (b)	2.47 ± 0.12 (a)
	Zn	0.46 ± 0.03 (b)	55.22 ± 2.10 (a)	4.39 ± 0.15 (b)	6.59 ± 0.18 (a)
	Mn	0.82 ± 0.05 (b)	13.31 ± 0.33 (a)	2.87 ± 0.08 (b)	3.25 ± 0.07 (a)
	Fe	2.14 ± 0.11 (b)	29.43 ± 1.76 (a)	6.17 ± 0.21 (b)	7.73 ± 0.12 (a)
	Li	0.34 ± 0.015 (b)	0.92 ± 0.05 (a)	2.03 ± 0.12 (b)	2.30 ± 0.01 (a)

Results are presented as mean values ± standard deviations (*n* ≥ 3); different letters within the same row indicate significant differences (*p <* 0.05) between mean values (Tukey test). 5-HMF is 5-hydroxymethylfurfural.

**Table 3:** Fatty acids profiles in white wheat flour (WWF), yellow mealworm powder (YMP), and selected biscuits.

Fatty Acid % of Total Fatty Acids ± SD					
Fatty Acid		White Wheat Flour WWF	Yellow Mealworm Powder YMP	Reference Biscuits (100% White Wheat Flour) 0% (R1)	Biscuits Containing 15% Yellow Mealworm Powder 15% (R3)
Caprylic	C8:0	0.35 ± 0.01	Nd	0.32 ± 0.05 (a)	0.19 ± 0.02 (b)
Capric	C10:0	0.42 ± 0.04	Nd	0.25 ± 0.03 (a)	0.23 ± 0.05 (a)
Lauric	C12:0	nd	0.21 ± 0.01	nd	0.03 ± 0.01
Myristic	C14:0	0.20 ± 0.01 (b)	1.91 ± 0.11 (a)	0.17 ± 0.02 (b)	0.25 ± 0.02 (a)
Pentadecanoic	C15:0	0.21 ± 0.02 (a)	0.18 ± 0.01 (b)	0.16 ± 0.06 (a)	0.13 ± 0.03 (a)
Palmitic	C16:0	14.24 ± 0.05 (b)	15.78 ± 0.22 (a)	12.15 ± 0.027 (a)	12.4 ± 0.21 (a)
Stearic	C18:0	1.92 ± 0.01 (b)	2.22 ± 0.06 (a)	2.53 ± 0.31 (a)	2.57 ± 0.32 (a)
Arachidic	C20:0	nd	0.1 ± 0.02	nd	0.11 ± 0.02
Behenic	C22:0	0.62 ± 0.03	Nd	0.47 ± 0.02 (a)	0.39 ± 0.04 (b)
Myristoleic	C14:1, *n*-5	nd	0.06 ± 0.03	nd	nd
Palmitoleic	C16:1, *n*-7	0.50 ± 0.01 (b)	2.11 ± 0.03 (a)	0.38 ± 0.07 (a)	0.43 ± 0.01 (a)
Oleic	C18:1, *n*-9	16.65 ± 0.02 (b)	28.13 ± 0.12 (a)	19.3 ± 0.43 (b)	20.14 ± 0.22 (a)
Gondoic	C20:1, *n*-9	0.75 ± 0.01 (a)	0.19 ± 0.01 (b)	0.56 ± 0.03 (a)	0.37 ± 0.07 (b)
Linoleic	C18:2, *n-6*	61.47 ± 0.41 (a)	38.90 ± 0.13 (b)	60.11 ± 0.87 (a)	58.72 ± 0.93 (a)
Linolenic	C18:3, *n*-3	2.67 ± 0.01 (b)	10.21 ± 0.12 (a)	3.6 ± 0.19 (b)	4.04 ± 0.06 (a)
ΣSFA		17.96 ± 0.13 (b)	20.40 ± 0.19 (a)	15.58 ± 0.41 (a)	16.3 ± 0.47 (a)
ΣMUFA		17.90 ± 0.24 (b)	30.49 ± 0.05 (a)	20.24 ± 0.68 (a)	20.94 ± 0.35 (a)
ΣPUFA		64.14 ± 1.52 (a)	49.11 ± 0.34 (b)	63.71 ± 1.52 (a)	62.76 ± 2.54 (a)
*n-6/n-3* ratio		23.02 ± 0.26 (a)	3.81 ± 0.07 (b)	16.70 ± 0.36 (a)	14.53 ± 0.48 (b)
PUFA/SFA ratio		3.57	2.41	4.09	3.85
AI index		-	-	0.15	0.16
TI index		-	-	0.21	0.29
h/H index		-	-	6.74	6.55

Results are presented as means ± standard deviations of triplicate independent experiments (*n* ≥ 3); nd—not detected; SFA—saturated fatty acids; MUFA—monounsaturated fatty acids; PUFA—polyunsaturated fatty acids. AI (atherogenic index) = (C12:0 + 4 × C14:0 + C16:0)/(Σ*n*-6 + Σ*n*-3 + ΣMUFA); TI (thrombogenic index) = (C14:0 + C16:0 + C18:0)/[0.5 × ΣMUFA + 0.5 × Σ*n*-6 + 3 × Σ*n*-3 + (Σ*n*-3/Σ*n*-6)]; h/H (hypocholesterolemic/hypercholesterolemic ratio) = (C18:1 + C18:2 + C18:3 + C20:2 + C20:4 + C20:5 + C22:5 + C22:6)/(C14:0 + C16:0); different letters within the same row indicate significant differences (*p <* 0.05) between mean values (Tukey test).

**Table 4:** Amino acids profiles in white wheat flour (WWF), yellow mealworm powder (YMP), and selected biscuits.

Amino Acid % of Total Amino Acids ± SD				
Amino Acid	White Wheat Flour WWF	Yellow Mealworm Powder YMP	Reference Biscuits (100% White Wheat Flour) 0% (R1)	Biscuits Containing 15% Yellow Mealworm Powder 15% (R3)
Essential amino acids (EAAs)% of total amino acids				
Valine	7.34 ± 0.34 (b)	9.11 ± 1.04 (a)	5.02 ± 0.24 (b)	5.97 ± 0.26 (a)
Leucine	15.18 ± 0.43 (b)	18.53 ± 0.22 (a)	8.03 ± 0.62 (a)	8.87 ± 0.32 (a)
Isoleucine	8.04 ± 0.49 (a)	8.83 ± 1.10 (a)	6.34 ± 0.34 (a)	6.35 ± 0.74 (a)
Methionine	0.25 ± 0.11 (b)	0.45 ± 0.11 (a)	4.23 ± 0.25 (a)	4.14 ± 0.43 (a)
Threonine	1.02 ± 0.87 (a)	1.24 ± 0.34 (a)	4.56 ± 0.17 (a)	5.02 ± 0362 (a)
Phenylalanine	5.03 ± 0.93 (b)	6.19 ± 0.12 (a)	5.31 ± 0.66 (a)	6.11 ± 0.44 (a)
Lysine	0.06 ± 0.01 (b)	0.21 ± 0.01 (a)	4.46 ± 0.57 (a)	5.21 ± 0.34 (a)
Histidine	nd	nd	nd	nd
Tyrosine	2.53 ± 1.03 (b)	11.92 ± 0.64 (a)	7.12 ± 0.90 (a)	8.64 ± 1.92 (a)
Total EAAs	39.45 ± 0.41 (b)	56.48 ± 3.59 (a)	45.07 ± 087 (b)	50.31 ± 1.14 (a)
Nonessential amino acids (NEAAs)% of total amino acids				
Alanine	6.32 ± 1.12 (b)	7.78 ± 0.09 (a)	4.39 ± 0.17 (a)	4.34 ± 0.21 (a)
Glycine	2.68 ± 0.22 (a)	3.35 ± 0.59 (a)	3.32 ± 0.08 (b)	3.76 ± 0.34 (a)
Proline	14.06 ± 0.21 (a)	5.01 ± 0.43 (b)	21.27 ± 0.74 (a)	17.11 ± 0.77 (b)
Serine	3.44 ± 1.27 (a)	2.12 ± 0.87 (a)	3.82 ± 0.31 (a)	3.82 ± 0.26 (a)
Aspartic acid	2.82 ± 0.10 (a)	3.67 ± 0.96 (a)	1.85 ± 0.34 (a)	1.83 ± 0.08 (a)
Hydroxyproline	0.02 ± 0.01 (b)	0.12 ± 0.01 (a)	nd	nd
Glutamic acid	28.01 ± 0.02 (a)	4.75 ± 1.34 (b)	14.4 ± 0.65 (a)	11.22 ± 0.18 (b)
Asparagine	nd	nd	1.86 ± 0.07 (a)	1.52 ± 0.03 (b)
Glutamine	nd	nd	nd	nd
Arginine	3.20 ± 0.16 (b)	16.72 ± 0.44 (a)	4.02 ± 0.09 (b)	6.09 ± 0.04 (a)
Total NEAAs	60.55 ± 0.28 (a)	43.52 ± 5.72 (b)	54.93 ± 1.48 (a)	49.69 ± 0.11 (b)

Results are presented as mean values ± standard deviations (*n* ≥ 3); different letters within the same row indicate significant differences (*p <* 0.05) between mean values (determined via Tukey test).

**Table 5:** The parameters associated with the four directions considered to characterise the first replica of starch digestion in R1 biscuits, which were extracted from LOS mathematical model described in the Figure 2b.

LOS Model	Black Line in Figure 2b	Blue Line in Figure 2b	Red Line in Figure 2b
1. The linear regression model			
n	21	6	15
a	0.5192	2.0225	0.1061
b	−0.0331	−0.1262	−0.0296
R	−0.9090	−0.9798	−0.8422
R^2^	0.8263	0.9600	0.7094
Adj R^2^	0.8171	0.9499	0.6870
SEE	0.8762	0.2696	0.8798
2. Analysis of variance			
SS reg	69.3702	6.9688	24.5623
*df* reg	1	1	1
MS reg	69.3702	6.9688	24.5623
SS error	14.5874	0.2907	10.0625
*df* error	19	4	13
MS error	0.7678	0.0727	0.7740
F	90.3543	95.8752	31.7326
p	1.1837 × 10 ^−8^	6.0972 × 10 ^−4^	8.1570 × 10 ^−5^
3. Statistics of regression model coefficients			
Sy/x	0.8762	0.2696	0.8798
Sa	0.3361	46.5344	0.5970
ta	1.5445	0.0435	0.1777
pa	0.1390	0.9674	0.8617
Sb	0.0035	2.6960	0.0053
tb	−9.5055	−0.0468	−5.6332
pb	1.1837 × 10 ^−8^	9.6491 × 10 ^−1^	8.1570 × 10 ^−5^
4. The coordinates of the intersection point			
		x [min]	y [ln(%/min)]
[d1] ∩ [d2]		19.8411	2.0162

n—the number of value pairs; a—intercept of the linear regression model; b—slope; R—correlation coefficient; R^2^—coefficient of determination; Adj R^2^—adjusted R^2^; SEE—standard errors of estimate; SS reg—sum-of-squares regression; df reg—degree of freedom of regression; MS reg—mean squares of regression; SS error—sum-of-square errors; df error—degree-of-freedom errors; MS error—mean square errors; F—Fisher factor; p—significance level of regression model; Sy/x—standard deviation of the residuals; Sa—deviation of intercept; ta—Student parameter of intercept; pa—level of probability associated with intercept; Sb—deviation of slope; tb—Student parameter of slope; pb—level of probability for slope; [d1] ∩ [d2]—intersection point of d1 and d2 lines.

**Table 6:** Average values of the parameters associated with kinetic equations describing the digestion of RDS and SDS fractions subjected to application of LOS and NLLS kinetic models.

	Reference Biscuits (100% White Wheat Flour) 0% (R1)	Biscuits Containing 15% Yellow Mealworm Powder 15% (R3)
LOS model		
K_RDS_, min^−1^	0.12 ± 0.01 (a)	0.09 ± 0.01 (b)
K_SDS_, min^−1^	0.02 ± 0.01 (a)	0.01 ± 0.00 (a)
ts, min	28.29 ± 7.40 (a)	25.99 ± 4.37 (a)
NLLS model		
C_RDS∞_, %	61.02 ± 2.84 (a)	45.73 ± 2.19 (b)
C_SDS∞_, %	21.15 ± 5.35 (b)	32.45 ± 0.83 (a)
K_RDS_, min^−1^	0.14 ± 0.01 (a)	0.13 ± 0.01 (a)
K_SDS_, min^−1^	0.01 ± 0.01 (a)	0.01 ± 0.00 (a)
ts, min	26.71 ± 7.01 (a)	22.87 ± 0.56 (a)

Different letters within the same row indicate significant differences (*p <* 0.05) between mean values (via Tukey test); NLLS—non-linear least squares model; LOS—the logarithm of slope model; K_RDS_—the RDS digestion rate coefficient; K_SDS_—the SDS digestion rate coefficient; CRDS_∞_—the ratio of digested RDS at infinite reaction time; CSDS_∞_—the ratio of digested SDS at infinite reaction time; ts—the starting time for the digestion of the SDS fraction; SDS—slowly digestible starch; RDS—rapidly digestible starch.

## Data Availability

Data is unavailable due to privacy.

## Supplementary Materials

The following supporting information can be downloaded at: https://www.mdpi.com/article/10.3390/nu15040997/s1.
