# Supplementary material for: Yellow Mealworm (Tenebrio molitor) Powder Promotes a High Bioaccessible Protein Fraction and Low Glycaemic Index in Biscuits
Source: Nutrients. 2023 Feb 16;15(4):997. doi: 10.3390/nu15040997 (PMC9961205; doi:10.3390/nu15040997)
Supplement: Supplementary file 1 [file nutrients-15-00997-s001.zip › nutrients-2091795-supplementary.pdf]

Yellow Mealworm (*Tenebrio molitor*) Powder Promotes a High Bioaccessible Protein Fraction and Low Glycaemic Index in Biscuits

Anca Mihaly Cozmuta[1*], Alexandra Uivarasan[1], Anca Peter[1], Camelia Nicula[1], Dalma Emoke Kovacs[2] and Leonard Mihaly Cozmuta[1*]

[1] Technical University of Cluj Napoca, North University Center of Baia Mare, Victoriei Str. 76, Baia Mare, Romania
[2] Research Institute for Analytical Instrumentation, INCDO INOE 2000, Donath 63, 400293, Cluj Napoca, Romania
*Correspondence: AMC: ancamihalycozmuta@gmail.com; LMC: mihalyleonard@yahoo.com

2.SM. Materials and methods

Total lipid content was determined by Soxhlet extraction (Velp Scientifica 148 Italy, method 920.39 - AOAC, 1995). Accurate weighed 2 g of sample was subjected to preliminary acid hydrolysis with 10 mL of HCl at 80°C for 40 minutes. The lipids were extracted for 2 hrs in petroleum ether. After solvent evaporation, the total lipid content was calculated on gravimetric basis and expressed as g of lipids/100 g of dry sample.

The **crude fibres** in samples were quantified according to the method proposed by Commission Regulation (EC) No. 152/2009. It involves the acid hydrolysis of 1 g o sample with 150 mL 0.13 mol/L H_2SO_4 at boiling point for 5 minutes for the extraction of sugars and starch, followed by alkaline hydrolysis with 150 mL 0.23 mol/L KOH to remove proteins and some hemi-cellulose and lignin. The residue is filtrated, dried, weighed and ashed at 500°C for at 1 hr. The crude fibre content is calculated according to the expression:

$$\text{Crude fibre (g/100 g)} = (m_0 - m_1)*100/m \qquad \text{(Eq. 1.SM)}$$

where: m is the weight of sample, g; m_0 is the loss of weight after washing, g; m_1 is the loss of weight after ashing during the blank test, g.

Minerals analysis involved a microwave digestion of 0.3 g of sample in the presence of 6 mL 67% HNO_3 and 1 mL 37% HCl for 5 minutes at 145°C and 60% power, 10 minutes at 170°C and15 minutes at 200°C at 80% power (Berghof, MWS-2). The resulted solution was diluted to 100 mL with 67% HNO_3 and submitted to Perkin Elmer AAnalyst 800 spectrometer (Perkin Elmer, USA) for the quantification of the minerals content. The results are expressed as mg of mineral/100 g of dry sample.

Fatty acids profile involves two steps: the extraction and derivatization of fatty acids and their identification and dosing (Dehelean et al., 2019).

a)Extraction and derivatisation of fatty acids

A known amount of the sample was put into contact with 50 mL isooctane and 2.5 mL methanolic solution of KOH, and the resulting solution was homogenized in the ultrasound bath at 80°C for 20 minutes. The filtered solution was neutralized with 1 g $NaHSO_4$ x H_2O.

b) Identification and dosing of fatty acids

1 µL of the neutralized solution was introduced into a FAME gas chromatograph - Gas chromatograph with flame ionization detector GC-FID 7890A Agilent equipped with db-wax silica capillary column (30 m x 0.25 mm i.d. x 0.25 µm film thickness). The working conditions were as follows: the injector temperature of 250°C, He gas as a carrier with the flow rate of 1 $mL\cdot min^{-1}$, the column temperature from 100 to 180°C with a speed of 7°C/min with 5 minutes isothermal regime, followed by the increase from 180 to 240°C at a speed of 10 °C/min and another 10 minutes of isothermal regime.

Amino acids profile also involves two stages: the extraction and derivatization of amino acids and their identification and dosing.

a)Extraction of amino acids and derivatisation of amino acids

1 g of the sample was ground and vigorously mixed with 5 mL of distilled water. After centrifuging for 5 minutes, 0.5 mL of supernatant was harvested and passed through a Dowex 50W-W8 ion exchange resin, and subsequently eluted with a 4M NH_4OH solution. Derivatization was done in two steps: esterification of the extracted amino acids with a mixture consisting of butanol-acetyl chloride (4:1 v/v) for 1h at 100°C, and acetylation with 100 µl of trifloractic anhydride at 60°C for 30 min.

b)Identification and dosing of amino acids

1 µL of the solution of the derived amino acids was separated into a chromatograph gas with a flame ionization detector GC-FID Trace GC1310 Thermo Scientific, using a capillary column Rtx-5MS capillary 30 mm x 0.25mm, at a film thickness of 0.25 µm, at a schedule of temperature increase from 50°C (1 min) by 10°C$\cdot min^{-1}$ to 100°C, 4°C$\cdot min^{-1}$ at 200°C and 20°oC$\cdot min^{-1}$ at 290°oC (maintained for 5 min). The injector was kept at 250°C and the detector at 280°C. The carrier gas was He, with a flow rate of 1 $ml\cdot min^{-1.}$.

Determination of HMF amount

Extraction and analysis of HMF were performed by the method described by Delgado-Andrade et al. (2010) with some modifications. 1 g of homogenized sample was weighted in a 40 mL centrifuge tube and 19 mL distilled water was added. Samples were vortexed for 1 min (1600 rpm), 0.5 mL Carrez I solution was added and the mixture was vortexed for 10 seconds, 0.5 mL Carrez II solution was added and the mixture was vortexed for 10 seconds. The tubes were then centrifuged at 14,000 g at 4°C for 10 min. Supernatants were filtered (Whatman N. 4), then microfiltered (0.45 µm acetate filters). The supernatant was mixed with 5 ml of 10% (w/v) p-toluidine solution and 1 ml of 0.5% (w/v) barbituric acid was added. After 3 min, the absorbance reading at 550 nm was taken against the blank sample. The HMF concentration was calculated using the calibration curve. The results were expressed as mg/Kg.

In vitro digestion study

In vitro digestion of selected biscuits was carried out in triplicate considering oral, gastric and intestinal stages according to the INFOGEST protocol described by Brodkorb et al. (2019). The composition of simulated salivary fluid (SFF), simulated gastric fluid (SGF), simulated intestinal fluid (SIF) and enzymes solutions are depicted in the Table 1.SM. The next steps were followed according to the protocol described by Brodkorb et al. (2019):

Oral phase

1. The SSF is pre-wormed on water bath at 7°C

2. 5 g of bread previous minced to simulate mastication is diluted with 4 mL SSF until a swallowable bolus with a paste-like (mustard or tomato) consistency is obtained. If it is thicker than such a paste small amount of water is added to achieve the proper consistency (the final ratio bread:SSF 1:1 wt:wt).

3. Measure the volume of the final digestion mixture of the food + SSF mixture to be further used.

4. Add 0.025 mL $CaCl_2 \cdot 2H_2O$.

5. Add 0.75 mL salivary amylase to achieve an activity of 75 U/mL in the final mixture.

6. Add the remaining water (0.225 mL) in case that it was not used at step 2.

7. Incubate the bolus while mixing for 2 min at 37 °C.

Gastric phase (2–3 h)

1. Pre-warm the SGF electrolyte stock solution at 37 °C. Add SGF electrolyte stock solution to the oral bolus to achieve a final ratio of 1:1 (vol/vol).

2. Adjust the pH to 3.0 by adding a defined volume of HCl previously determined during a pH-test
adjustment experiment.

3. Add CaCl2(H2O)2 solution in order to achieve a final concentration of 0.15 mM in SGF.

4. Add the porcine pepsin solution prepared in water to achieve an activity of 2,000 U/mL in the final
digestion mixture.

5. Add the gastric lipase solution prepared in water to achieve an activity of 60 U/mL in the final
digestion mixture.

6. Verify the pH and adjust to 3.0 if necessary.

7. Add water in order to achieve a 1× concentration of SGF.

8. Incubate the samples at 37 °C, mixing the digestive mixture sufficiently (e.g., rotating wheel,
shaking incubator) for 2 h from the point at which pepsin was added.

Intestinal phase (2–3 h)

1. Pre-warm the SIF electrolyte stock solution in a 37 °C water bath. Add SIF electrolyte to the gastric chyme to achieve a final ratio of 1:1 (vol/vol).

2. Adjust the pH to 7.0 by adding a defined volume of NaOH previously determined during a pH-test

adjustment experiment.

3. Add the bile solution to the SIF/gastric chime solution in order to reach a final concentration of 10 mM. Place the solution in a rotating wheel mixer at 37 °C for at least 30 min to achieve complete bile solubilization.

4. Add CaCl2(H2O)2 solution in order to reach a concentration of 0.6 mM in SIF.

5. Add the pancreatin suspension in SIF solution to achieve a trypsin activity of 100 U/mL in the final mixture. Additional pancreatic lipase may be needed for the digestion of fat-containing food to reach the required lipase activity to achieve a lipase activity of 2,000 U/mL in the final mixture.

6. Verify the pH and adjust to 7.0 if necessary.

7. Add water in order to achieve a 1× concentration of the SIF.

8. Incubate the samples at 37 °C, using a rotating wheel or shaking incubator to mix the digestive mixture sufficiently for 2 h, starting at the point when pancreatic enzymes were added.

Table S1. The composition of simulated salivary fluid (SFF), simulated gastric fluid (SGF), simulated intestinal fluid (SIF) and enzymes solutions used in the *in vitro* digestion (Brodkorb et al. (2019).

Reagent	Concentration of stock solution, g/L	Simulating salivary fluid (SSF, pH = 7)	Simulating gastric fluid (SGF, pH = 3)	Simulating intestinal fluid (SIF, pH = 7)
		The volume of the stock solution used for 400 mL of fluid diluted with distilled water, mL	The volume of the stock solution used for 400 mL of fluid diluted with distilled water, mL	The volume of the stock solution used for 400 mL of fluid diluted with distilled water, mL
KCl	37.3	15.1	6.9	6.8
KH_2PO_4	68	3.7	0.9	0.8
NaHCO3	84	6.8	12.5	42.5
NaCl	117	-	11.8	9.6
$MgCl_2·6H_2O$	30.5	0.5	0.4	1.1
$(NH_4)_2CO_3$	48	0.06	0.5	-
HCl	-	1.1	1.3	0.7
$CaCl_2·2H_2O$*	44.1	0.025	0.005	0.04
Salivary amylase (for starch-containing food)	10 mg/mL	-	-	-
Pepsin	20 mg/mL	-	-	-
Gastric lipase	100 mg/mL	-	-	-
Bile salts	200 mg/mL	-	-	-
Trypsin in pancreatin	133.3 mL	-	-	-

*$CaCl_2·2H_2O$ – should be added immediately before use to void precipitation

References

AOAC 920.39.B; Determination of Total Fat in Flour, Bread, Bakery Products and Pasta with Preliminary Acid Hydrolysis, Velp Scientifica—SR 148 Manual, 45. AOAC International, 2009.

Berghof, MWS-2. Microwave Pressure Digestion Food, Pharma, Cosmetics. 6.0. Application Report Microwave Pressure Digestion, speed wave MWS-2. Berghof Products + Instruments GmbH, Eningen, Germany.

Besir A., Yazici F., Mortas M., Gul O. (2021). A novel spectrophotometric method based on Seliwanoff test to determine 5-(Hydroxymethyl) furfural (HMF) in honey: Development, in house validation and application. LWT, 139, 110602.

Brodkorb, A., Balance, S., Bohn, T., Bourlieu-Lacanal, C., Boutrou, R., Carrière, F., Clemente, A., Corredig, M., Dupont, D., Dufour, C., Edwards, C., Golding, M., Karakaya, S., Kirkhus, B., Le Feunteun, S., Lesmes, U., Macierzanka, A., Mackie, A.,R., Martins, C., Marze, S., McClements, D.J., Ménard, O., Minekus, M., Portmann, R., Santos, C.,N., Souchon, I., Singh, R.,P., Vegarud, G.,E., Wickham, M.,S.,J, Weitschies, W., Recio, I. (2019). INFOGEST static in vitro simulation of gastrointestinal food digestion. Nature Protocols, 14, 991 – 1014.

Commission Regulation (EC) 152/2009 laying down the methods of sampling and analysis for the official control of feed.

Dehelean A, Kovacs ED, Magdas DA, Cristea G, Voica C, Feher I, Kovacs MH, Marincas O, Zhang Z, Li P, Zang Q (2019). Infant supplement quality evaluation by inductively coupled plasma-mass spectrometry, gas chromatography, and isotope ratio mass spectrometry. Analytical Letters, 52(1), 127-138.

Delgado-Andrade C., Seiquer I., Haro A., Castellano R., Navarro M.P. (2010). Development of the Maillard reaction in foods cooked by different techniques. Intake of Maillard-derived compounds. Food Chemistry, 122, 145–153.
